# Synthesis and Antiviral Activity of 5‑(4‑Chlorophenyl)-1,3,4-Thiadiazole Sulfonamides 

**DOI:** 10.3390/molecules15129046

**Published:** 2010-12-09

**Authors:** Zhuo Chen, Weiming Xu, Keming Liu, Song Yang, Huitao Fan, Pinaki S. Bhadury, De-Yu Hu, Yuping Zhang

**Affiliations:** State Key Laboratory Breeding Base of Green Pesticide and Agricultural Bioengineering, Key Laboratory of Green Pesticide and Agricultural Bioengineering, Ministry of Education, Guizhou University, Guiyang 550025, China; Email: gychecnzhuo@yahoo.com.cn (Z.C.); xuweiming2009@163.com (W.X.); lkmingsd@126.com (K.L.); fanhuitao0818@163.com (H.F.); bhadury@gzu.edu.cn (P.S.B.); zhangyupinggz@163.com (Y.Z.)

**Keywords:** sulfonamide derivatives, 1,3,4-thiadiazole moiety, synthesis, antiviral activity

## Abstract

Starting from 4-chlorobenzoic acid, 10 new 5-(4-chlorophenyl)-*N*-substituted-*N*-1,3,4-thiadiazole-2-sulfonamide derivatives were synthesized in six-steps. Esterification of 4-chlorobenzoic acid with methanol and subsequent hydrazination, salt formation and cyclization afforded 5-(4-chlorophen-yl)-1,3,4-thiadiazole-2-thiol (**5**). Conversion of this intermediate into sulfonyl chloride **6**, followed by nucleophilic attack of the amines gave the title sulfonamides **7a-7j **whose structures were confirmed by NMR, IR and elemental analysis. The bioassay tests showed that compounds **7b **and **7i **possessed certain anti-tobacco mosaic virus activity.

## 1. Introduction

Sulfonamide drugs which have brought about an antibiotic revolution in medicine are associated with a wide range of biological activities [1,2,3]. Sulfonamide derivatives have also been reported to possess antifungal [4,5,6] and herbicidal [7] properties for potential agricultural applications.

**Figure 1:**
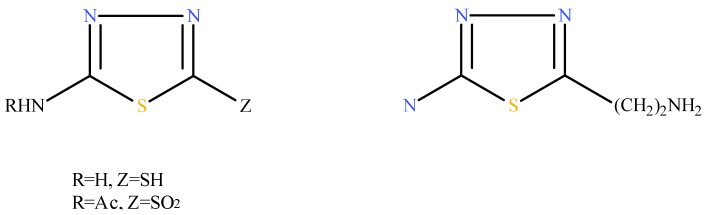
Representative structures of some potent 1,3,4-thiadiazoles.

On the other hand, certain 1,3,4-thiadiazoles prepared by different groups have often displayed other interesting bioactivities, e.g. anticonvulsant [8], antifungal and antibacterial properties [9,10,11]. Some representative examples of these derivatives possessing potent activity are shown in Figure 1. 

In our previous work, we synthesized a series of 2-alkylthio-5-(3,4,5-trimethoxyphenyl)-1,3,4-thiadiazole derivatives to inhibit the growth of tumor cells [12]. We also obtained some chiral thioureas and α-aminophosphonates, cyanoacrylates and certain heterocyclic compounds with the aim of protecting tobacco plants affected with tobacco mosaic virus (TMV), but barring a few exceptional cases, these compounds did not reveal the expected anti-TMV activity [13]. Since the incorporation of sulfonamides into 1,3,4-thiadiazole rings can produce compounds that can act as carbonic anhydrase inhibitors [3], herein we have combined the two pharmacophoric units into one structure to generate new compounds with potential plant anti-viral activities. Thus, in the present investigation, 10 novel sulfonamide derivatives containing 1,3,4-thiadiazole rings were synthesized and screened for their anti-TMV activities. The structures of the synthesized products were confirmed by IR, ^1^H-NMR and ^13^C-NMR spectral data and elemental analysis. The bioactivity study results revealed that some of the prepared compounds indeed possessed a certain degree of antiviral activity against TMV. Among them, compounds **7b **and **7i** exhibited approximately 50% TMV inhibition, in the same range as that displayed by the commercial antifungal ningnanmycin. 

## 2. Results and Discussion

### 2.1. Chemistry

**Scheme 1:**
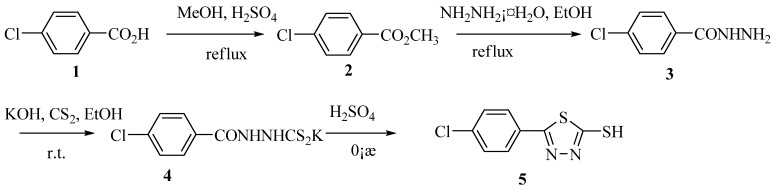
Synthetic route to intermediate **5**.

**Scheme 2:**
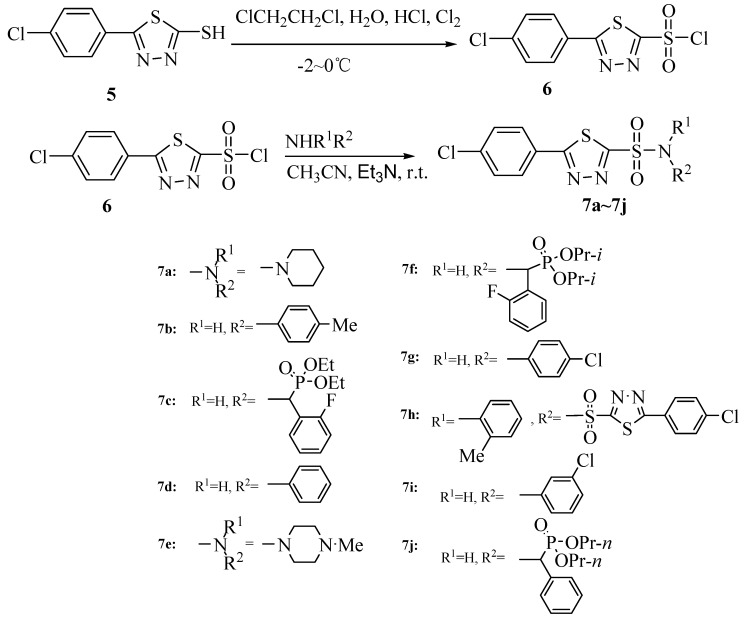
Synthetic route to title compounds **7a-7j**.

The synthetic routes to the intermediate **5** and title compounds **7a-7j **are shown in Scheme 1 and Scheme 2 respectively. As shown in Scheme 1, the starting material 4-chlorobenzoic acid (**1**) was easily converted into methyl benzoate **2** in 80% yield by methanol in the presence of concentrated sulfuric acid. The methyl ester was then treated with hydrazine hydrate in ethanol to afford a 90% yield of compound **3**, which on treatment with KOH and CS_2_ in ethanol at ambient temperature gave the corresponding potassium salt **4 **in 94% yield. Finally, cyclization of **4** in acidic medium at 0 °C for 6 h produced the thiol intermediate **5**. The preparation of the next key intermediate, the sulfonyl chloride **6**, from thiol **5** by treatment with chlorine was then optimized in different solvents, e.g. acetic acid/water, methylene chloride/water and 1,2-dichloroethane/water. The poor solubility of the starting material thiol and the product sulfonyl chloride in acetic acid/water and ready dissolution of both the components in methylene chloride/water rendered these solvents ineffective to monitor the progress of the sulfonylation reaction.

On the other hand, the poor solubility of the thiol and complete miscibility of sulfonyl chloride in 1,2-dichloroethane/water were crucial to follow the reaction to its completion. Thus, under optimal conditions, a suspension of the thiol in 1,2-dichloroethane/water was bubbled with chlorine gas under constant stirring at low temperature (Scheme 2). When the color of the reaction system turned yellowish green, the gas flow was discontinued, the organic phase was separated, dried on anhydrous magnesium sulfate and the solvent removed to afford the desired product. The title compounds **7a-7j** were then prepared by the reaction of nucleophilic amines with the intermediate **6**, as shown in Scheme 2. The role of various solvents, reaction temperatures and acid scavengers was studied for optimization of the reaction conditions using compound **7b** as the model. It is evident from the data presented in Table 1 that out of the three solvents acetonitrile, dichloromethane and acetone selected for the study, the first one (entry 1) afforded the best result in terms of chemical yield. Having established acetonitrile as the ideal solvent for the reaction, the effect of two different organic bases, e.g. triethylamine and pyridine was investigated. While triethylamine showed significant improvement compared to the control, pyridine displayed much inferior results. With regard to the reaction temperature (Table 2) for the model reaction with *p*-toluidine serving as the reacting amine, the highest yield of the product **7b** was recorded at room temperature (entry 2) compared to the one conducted at a lower or higher temperature (entries 1 and 3). The reaction appears to be relatively sluggish at 10 °C and might produce some side products at 40 °C or higher under the influence of the basic catalyst.

Therefore, under optimal conditions, the sulfonyl chloride **6** was reacted with the different amines in acetonitrile at room temperature in the presence of triethylamine which was used as an acid scavenger for the liberated HCl.

**Table 1:** The effect of solvent on the yield of compound **7b**.

Entry	Solvent	Time (h)	t (°C)	Yield (%)
1	Acetonitrile	5	25	32.8
2	Dichloromethane	5	25	17.5
3	Acetone	5	25	trace

The structures of the title compounds **7a-7j **were unequivocally characterized by spectral data and elemental analyses. The existence of characteristic IR absorption in the 1,600–1,550 cm^−1^ region was indicative of a C=N group and the two strong absorption bands around 1,160~1,190 cm^−1^ and 1,340~1,380 cm^−1^ respectively confirmed the presence of a sulfonyl functionality. In the ^1^H-NMR spectra of the title compounds, the D_2_O exchangeable sulfonamide proton appeared at 10–11 ppm, whereas the two different types of aromatic protons of the 4-chlorophenyl ring showed up as two distinct doublets at 7.5–8.3 ppm with coupling constants of 7.5 and 8.6Hz, respectively.

**Table 2:** The effect of temperature on the yield of compound **7b**.

Entry	Solvent	Acid acceptor	t	Time	Yield
	(V)	(V)	(°C)	(h)	(%)
1	Acetonitrile	Triethylamine	10	5	19.2
	(15 mL)	(0.15 mL)			
2	Acetonitrile	Triethylamine	r. t.	5	48.0
	(15 mL)	(0.15 mL)			
3	Acetonitrile	Triethylamine	40	5	41.0
	(15 mL)	(0.15 mL)			

### 2.2. Antiviral activity bioassay

The newly synthesized derivatives were evaluated for their antiviral activity against tobacco mosaic virus by the half leaf method. The bioassay results as obtained at 500 μg/mL using ningnanmycin as the control are shown in Table 3. It is clear that the title compounds **7a-7j** showed a certain degree of antiviral activity against tobacco mosaic virus. The structural modification caused by changing the substituents (R^1^ and R^2^) in the sulfonamide moiety has a wide impact on anti-viral activity of the prepared compounds. Thus, amongst the compounds **7c**, **7f** and **7j** bearing an α-aminophosphate moiety, the one derived from diisopropyl phosphite and containing a 2-fluorophenyl group at the α-carbon of the phosphonate (**7f**) exhibited better TMV inhibition. Nevertheless, these compounds were found to be less potent than their counterparts **7b** and **7i** where the phosphonate moiety was replaced by a 4-methylphenyl or 3-chlorophenyl group, respectively. The importance of these suitably substituted aryl substituents (for **7b** and **7i**) was evident when their inhibitory activity was compared with that of **7d** with an unsubstituted phenyl ring, which showed much lower activity. Interestingly, the title compound **7a** bearing a heterocyclic ring substituent in the sulfonamide part displayed reasonably good activity. 

**Table 3:** The inhibitory activity of compounds **7a~7j** to TMV.

Compd	Concentration (mg/mL )	Inhibition rate（%）
**7a**	0.5	38.42
**7b**	0.5	42.00
**7c**	0.5	31.55
**7d**	0.5	34.70
**7e**	0.5	25.00
**7f**	0.5	39.60
**7g**	0.5	31.60
**7h**	0.5	36.80
**7i**	0.5	42.49
**7j**	0.5	33.92
Ningnanmycin	0.5	54.51

## 3. Experimental

### 3.1. General

Melting points were determined on an X-4 melting point instrument. The ^1^H- and ^13^C NMR spectra were recorded on a JOEL ECX-500 NMR spectrometer (operating at 500 and 125 MHz MHz frequency, respectively). Chemical shifts were reported in ppm from internal tetramethylsilane used as standard and are given in *δ*-units. The solvents used for the NMR spectra were DMSO-*d*_6 _or CDCl_3._ Infrared spectra were taken on an IR Prestige-21 Fourier Transform infrared spectrophotometer in potassium bromide pellets. Elemental analyses were performed on an Elementar Vario-III. All reactions were monitored by thin layer chromatography, carried out on 0.3 mm silica gel F-254 (Merck) plates using UV light ZF-2 (253.7 and 365.0 nm) for detection. Commercial chemicals and solvents were of reagent or analytical grade and used without further purification.

### 3.2. Preparation of methyl 4-chlorobenzoate (***2***)

A three-necked 250 mL flask fitted with a thermometer, dropping funnel and magnetic stirring bar was charged with 4-chlorobenzoic acid (40 g, 0.26 mol) and anhydrous methanol (100 mL). To this solution concentrated sulfuric acid (16 mL) was added dropwise through a dropping funnel. The solution was heated to reflux for 6 h, cooled to room temperature and poured into an equal volume of water. The organic phase was washed with saturated aqueous sodium bicarbonate solution (10 mL) to a pH of 7–8. The crude product was dried over magnesium sulfate and the yellow oil was used for the next step without further purification, yield 80%; ^1^H-NMR (CDCl_3_): *δ* 7.95 (d, 2H, ArH, *J* = 8.6 Hz), 7.39 (d, 2H, ArH, *J* = 8.6 Hz), 3.89 (s, 3H, CH_3_); ^13^C-NMR (CDCl_3_): *δ* 166.3, 139.4, 131.0, 128.8,52.3; IR: *ν* 3035 (CH-Ar), 3021 (CH-Ar), 2953 (CH_3_), 1726 (C=O), 1597, 1435, 1400, 1286, 1278, 1116, 1091, 900, 761 cm^−^^1^; Anal. Calcd. for C_8_H_7_ClO_2_: C 56.32%, H 4.14%. Found: C 56.17%, H 4.29%.

### 3.3. Preparation of 4-chlorobenzohydrazide (***3***)

A three-necked 250 mL flask fitted with a thermometer, dropping funnel and magnetic stirring bar was charged with **2** (37.6 g, 0.22 mol), anhydrous ethanol (80 mL) and hydrazine hydrate (40 mL, 80%). The solution was heated at reflux temperature for 8 h. The reaction mixture was cooled to 20 °C when a white solid precipitated. The crude product was recrystallized from anhydrous ethanol to yield 30.9 g of **3** as white crystals, yield 80%, m.p. 165–167 °C; ^1^H-NMR (CDCl_3_): *δ* 8.85 (s, 1H, NH), 7.70 (d, 2H, ArH, *J* = 5.2 Hz), 7.42 (d, 2H, ArH, *J* = 5.2 Hz), 4.10 (s, 2H, NH_2_); ^13^C-NMR (DMSO-*d*_6_): *δ* 165.3 (C=O), 136.4 (C=C), 132.5, 129.4, 129.0; IR: *ν* 3307 (NH asymmetric stretching), 3223 (NH asymmetric stretching), 3207 (NH), 3010 (CH-Ar), 1710 (CONH), 1640, 1560, 1346, 1095, 991, 883; Anal. Calcd. for C_7_H_7_ClN_2_O: C 49.28%, H 4.14%, N 16.42%. Found: C 49.36% H 4.10%, N 16.27%.

### 3.4. Potassium N, p-chlorobenzoylhydrazinodithio formate (***4***)

A three-necked 250 mL flask fitted with a thermometer, dropping funnel and a magnetic stir bar was charged with KOH (6.1 g, 0.11 mol), anhydrous ethanol (120 mL) and **3 **(14.6 g, 0.09 mol). The mixture was stirred to obtain a homogeneous solution and then CS_2_ (6.9 g) was slowly added through a dropping funnel. The reaction system was stirred at room temperature for an additional 6 h, filtered, and the resulting solid was used for the next step without further purification. White crystals, yield 94%, m.p. > 260 °C.

### 3.5. 5-(4--Chlorophenyl)-1,3,4-thiadiazole-2-thiol (***5***)

A three-necked 250 mL flask fitted with a thermometer and dropping funnel and magnetic stirring bar was first charged with sulfuric acid (80 mL, 98%) and then cooled on an ice bath with vigorous stirring. When the temperature reached below −2 °C, **4 **(22.9 g, 0.08 mol) was slowly added into it. After completion of the addition, the reaction mixture was kept below 0 °C and stirred for another 5 h. The mixture was poured into ice water (200 mL) whenupon a large amount of white solid precipitated which was separated by suction filtration. The solid was washed with water to a pH value of 6, the cake was dissolved in 10% aqueous NaOH and the insoluble part was removed through filtration. The filtrate was acidified by HCl (36%) to a pH of 2. The resulting white solid was filtered, washed with water and recrystallized from EtOH to yield 14.6g of **5** as white crystals, yield 81%, m.p. 198–200 °C; ^1^H-NMR (DMSO-*d_6_*): *δ* 7.78 (d, 2H, ArH, *J* = 8.6 Hz), 7.61 (d, 2H, ArH, *J* = 8.6 Hz); ^13^C-NMR (DMSO-*d*_6_): *δ* 188.2 (C-SH), 159.4 (C=N), 136.7(C=C), 130.0, 128.8, 127.9; IR: *ν* 3062 (CHAr), 1487, 1458, 1402, 1286, 1251 (N-N=C), 1124, 1087, 1066, 827; Anal. Calcd. for C_8_H_5_ClN_2_S_2_: C 42.01%, H 2.20%, N 12.25%. Found: C 42.03%, H 2.24%, N 12.56%.

### 3.6. 5-(4-Chlorophenyl)-1,3,4-thiadiazole-2-sulfonyl chloride (***6***)

A three-necked 50 mL flask fitted with a thermometer and magnetic stirring bar was successively charged with H_2_O (8 mL), 1,2-dichloroethane (15 mL) and **5 **(0.5 g, 2.2 mmol). The mixture was stirred on an ice bath and then hydrochloric acid (2 mL) was added into it. When the temperature reached −2 °C, the heterogeneous solution was slowly bubbled with chlorine gas. As soon as the color of the mixture turned yellowish green, the flow of gas was discontinued. The completion of the reaction was confirmed by TLC on silica gel (developing solvent: petroleum ether/ethyl acetate = 2:1, *v*/*v*) and then the reaction mixture was transferred to a separating funnel and the organic phase was separated, and the water solution was extracted with dichloromathane. The organic phase was dried over MgSO_4_ and the solvent was removed by rotary evaporation. The crude product **6** was purified by column chromatography on silica gel using a mixture of petroleum ether and ethyl acetate proportions (2:1, *V/V*) as the eluent to afford yellow crystals, yield 42%, m.p. 112–114 °C; ^1^H-NMR (CDCl_3_): *δ* 8.08 (d, 2H, ArH, *J* = 8.6 Hz), 7.67 (d, 2H, ArH, *J* = 8.6 Hz); ^13^C-NMR (CDCl_3_): *δ* 165.8 (C=N), 152.8, 138.9 (C=C), 129.7, 128.1, 121.4; IR: *ν* 3082 (CH-Ar), 1595, 1490, 1436, 1402, 1220, 1089, 1037, 1012, 972, 900, 835 cm^−1^.

### 3.7. 1-(5-(4-Chlorophenyl)-1,3,4-thiadiazol-2-ylsulfonyl)piperidine (***7a***)

To a stirred solution of piperidine (1.0 mmol) in acetonitrile (5 mL), were added **6 **(0.3 g, 1.0 mmol) and triethylamine (0.15 mL) at room temperature. The stirring was continued for another 6 hours. The solid was filtered under suction and dried. The pure compound was obtained by recrystallization from ethanol, white crystals, yield 26.5%, m.p. 198–200 °C; ^1^H-NMR (DMSO-d_6_): *δ* 8.11 (d, 2H, ArH, *J* = 8.6 Hz), 7.68 (d, 2H, ArH, *J* = 8.6 Hz), 3.27 (t, 4H, 2CH_2_, *J* = 5.1 Hz), 1.58–1.63 (m, 4H, 2CH_2_), 1.46–1.50 (m, 2H, CH_2_); ^13^C-NMR (DMSO-d_6_): *δ* 171.6 (C=N), 165.4, 137.8 (C=C), 130.6, 130.3, 127.8, 47.4, 25.2, 23.1; IR: 3088 (CH-Ar), 2954 (CH_2_), 2945, 2929, 2858, 1591, 1425, 1367, 1352, 1317, 1178, 1085, 941, 835, 715 cm^−1^; Anal. Calcd. for C_13_H_14_ClN_3_O_2_S_2_: C 45.41%, H 4.10%, N 12.22%. Found: C 45.46%, H 3.18, N 12.72%.

*5-(4-Chlorophenyl)-N-p-tolyl-1,3,4-thiadiazole-2-sulfonamide* (**7b**). To a stirred solution of *p*-toluidine (1.0 mmol) in acetonitrile (5 mL), were added **6 **(0.3 g, 1.0 mmol) and triethylamine (0.15 mL) at room temperature. The stirring was continued for another 6 hours. The solid was filtered under suction and dried. The pure compound was obtained by recrystallization from ethanol, white crystals, yield 37.8%, m.p. 223–225 °C; ^1^H-NMR (DMSO-d_6_): *δ* 7.90 (d, 2H, ArH, *J* = 5.1 Hz), 7.66 (d, 2H, ArH, *J* = 8.6 Hz), 7.51 (d, 2H, ArH, *J* = 8.7 Hz), 7.16 (d, 2H, ArH, *J* = 8.7 Hz), 2.68 (s, 3H, CH_3_); ^13^C- NMR (DMSO-*d*_6_): *δ* 160.7, 157.4, 131.4, 130.4, 129.8, 128.8, 127.8, 123.3, 120.9, 117.7, 20.9; IR: *ν* 3261 (NH), 3049 (CH-Ar), 2918 (CH_3_), 2858, 1614, 1602, 1573, 1485, 1402, 1093, 1051, 1012, 831, 819 cm^−1^; Anal. Calcd. for C_15_H_12_ClN_3_O_2_S_2_: C 49.24%, H 3.31%, N 11.49%. Found: C 49.58%, H 3.51%, N 11.43%. The compounds **7c-7j** were obtained by following a similar method of preparation. 

*Diethyl(2-(4-chlorophenyl)-1,3,4-thiadiazole-5-sulfonamido)(2-fluorophenyl)methylphosphonate* (**7c**). yield 11.2%, m.p. 203–205 °C; ^1^H-NMR (DMSO-*d*_6_): *δ* 10.40 (s, 1H, SO_2_NH), 7.93 (d, 2H, ArH, *J* = 8.6 Hz ), 7.64 (d, 2H, ArH, *J* = 7.5 Hz), 7.22 (dd, 1H, ArH, *J* = 8.6, 6.8 Hz ), 7.07 (t, 1H, ArH, *J* = 14.7, 8.5 Hz), 7.02 (t, 1H, ArH, *J* = 15.0 Hz), 5.18 (d, 1H, CH, *J* = 24.0 Hz), 4.02–4.10 (m, 2H, CH_2_), 3.86–3.94 (m, 1H, CH_2_), 3.75–3.83(m, 1H, CH_2_), 1.22 (t, 3H, CH_3_, *J* = 6.8 Hz), 1.03 (t, 3H, CH_3_, *J* = 14.5 Hz); ^13^C-NMR (DMSO-*d*_ 6_): *δ* 171.4, 167.9, 137.7, 130.7, 130.4, 130.2, 130.1, 127.6, 124.8, 115.6, 115.4, 63.8, 63.6, 48.3, 16.7, 16.4; IR: *ν* 3421 (NH), 3070 (CH-Ar), 2991 (CH_3_), 2893, 1494, 1458, 1357, 1284, 1248, 1165, 1112, 1093, 1074, 1053, 1031, 979, 837 cm^-1^; Anal. Calcd. for C_19_H_20_ClFN_3_O_5_PS_2_ : C 43.89%, H 3.88%, N 8.08. Found: C 43.72%, H 3.35%, N 8.18%.

*5-(4-Chlorophenyl)-N-phenyl-1,3,4-thiadiazole-2-sulfonamide* (**7d**). White crystals, yield 30%; m.p. 201–203 °C; ^1^H-NMR (DMSO-*d*_6_): *δ* 10.43 (s, 1H, NH), 8.05 (d, 2H, ArH, *J* = 8.6 Hz), 7.67 (d, 2H, ArH, *J* = 7.5 Hz), 7.34 (t, 2H, ArH, *J* = 18.3 Hz), 7.23 (d, 2H, ArH, *J* = 7.45 Hz), 7.18 (t, 1H, ArH, *J* = 14.3 Hz); ^13^C-NMR (DMSO-*d*_ 6_): *δ* 171.8, 167.3, 137.8, 136.4, 130.6, 130.2, 130.0, 127.6, 126.2, 122.2; IR: *ν* 3105 (NH), 3070 (CH-Ar), 2943, 2887, 2833, 1595, 1496, 1479, 1429, 1388, 1361, 1303, 1168, 1093, 1080, 1028, 1014, 937, 833 cm^−^^1^; Anal. Calcd. for C_14_H_10_ClN_3_O_2_S_2_: C 47.79%, H 2.86%, N 11.94%. Found: C 47.98%, H 2.15%, N 12.03%.

*1-(5-(4-Chlorophenyl)-1,3,4-thiadiazol-2-ylsulfonyl)-4-methylpiperazine* (**7e**). White powder, yield 38.4%, m.p. 172–173 °C; ^1^H-NMR (DMSO-*d*_6_): *δ* 8.12 (d, 2H, ArH, *J* = 8.6 Hz), 7.68 (d, 2H, ArH, *J* = 8.6 Hz), 3.30 (t, 4H, 2CH_2,_* J* = 4.6 Hz), 2.42 (t, 4H, 2CH_2_, *J* = 4.6 Hz), 2.19 (s, 3H, CH_3_); ^13^C-NMR (DMSO-*d*_ 6_): *δ* 171.7, 165.1,137.8, 130.6, 130.2, 127.8, 53.9, 46.5, 45.7; IR: *ν* 3109 (CH-Ar), 2956 (CH_3_), 2937, 2873, 2848, 1593, 1494, 1452, 1427, 1384, 1359, 1330, 1290, 1178, 1172, 1151, 1143, 1130, 1097, 1089, 1060, 979, 952, 831cm^-1^; Anal. Calcd. for C_13_H_15_ClN_4_O_2_S_2_: C 43.51%, H 4.21%, N 15.61. Found: C 43.54%, H 3.85%, N 15.95%.

*Diisopropyl(2-(4-chlorophenyl)-1,3,4-thiadiazole-5-sulfonamido)(2-fluorophenyl)* *methylphosphonate* (**7f**). White powder, yield 21%, m.p. 210–212 °C; ^1^H-NMR (DMSO-*d*_6_): *δ* 10.29 (s, 1H, NH), 7.90 (d, 2H, ArH, *J* = 8.6 Hz), 7.65 (d, 2H, ArH, *J* = 8.6 Hz), 7.47 (t, 1H, ArH, *J* = 7.45 Hz), 7.18 (q, 1H, ArH, *J* = 7.4 Hz), 7.08 (t, 1H, ArH, *J* = 8.6 Hz), 7.01 (t, 1H, ArH, *J* = 8.6 Hz), 5.07 (d, 1H, CH, *J* = 24.05 Hz), 4.62–4.68 (m, 1H, CH), 4.31–4.37 (m, 1H, CH), 1.26 (t, 6H, 2CH_3_, *J* = 6.3 Hz), 1.17 (d, 3H, CH_3_, *J* = 6.3 Hz), 0.85 (d, 3H, CH_3_, *J* = 6.3 Hz); ^13^C-NMR (DMSO-*d*_6_): *δ* 175.9, 171.3, 167.9, 158.8, 137.6, 130.3, 130.2, 127.6, 124.7, 115.4, 115.4, 115.4, 115.2, 72.4, 48.7, 47.4, 24.3, 24.2, 23.8, 23.2; IR: *ν* 3062 (NH), 3049 (CH-Ar), 2981 (CH ), 2877, 1595, 1494, 1456, 1421, 1379, 1359, 1244, 1230, 1168, 1093, 1004, 921, 842, 821cm^−^^1^; Anal. Calcd. for C_21_H_24_ClFN_3_O_5_PS_2_: C 46.03%, H 4.41%, N 7.67%. Found: C 45.64, H 3.83%, N 8.08%.

*N,5-bis(4-Chlorophenyl)-1,3,4-thiadiazole-2-sulfonamide* (**7g**). White powder, yield 30%, m.p. 245–247 °C; ^1^H-NMR (DMSO-*d*_6_): *δ* 8.07 (d, 2H, ArH, *J* = 8.6 Hz), 7.65 (d, 2H, ArH, *J* = 8.6 Hz), 7.41 (d, 2H, ArH, *J* = 8.6 Hz), 7.26 (d, 2H, ArH, *J* = 8.6 Hz); ^13^C-NMR (DMSO-*d*_6_): *δ* 171.9, 167.1, 130.6, 130.3, 130.2, 129.9, 127.6, 123.8; IR: *ν* 3093 (NH), 3045 (CH-Ar), 2912, 2872, 2819, 1489, 1431, 1404, 1363, 1182, 1172, 1093, 1012, 939, 840, 825 cm^−^^1^; Anal. Calcd. for C_14_H_9_Cl_2_N_3_O_2_S_2_: C 43.53%, H 2.35%, N 10.88%. Found: C 43.48%, H 2.65%, N 10.84%.

*1-(5-(4-Chlorophenyl)-1,3,4-thiadiazol-2-ylsulfonyl)-4-methylpiperazine* (**7h**). White powder, yield 21%, m.p. > 260 °C; ^1^H-NMR (DMSO-*d*_6_): *δ* 8.18 (d, 4H, ArH, *J* = 8.6 Hz), 7.73 (d, 4H, ArH, *J* = 8.6 Hz), 7.56–7.50 (m, 2H, ArH), 7.34–7.40 (m, 2H, ArH), 2.24 (s, 3H, 2CH_3_); ^13^C-NMR (DMSO-*d*_6_): *δ* 174.2, (C=N), 165.2, 138.5, 132.6, 131.8, 130.9, 130.6, 130.4, 130.2, 130.0, 127.3, 18.3; IR:*ν* 3089 (CH-Ar), 3053, 2880 (CH_3_), 1593, 1489, 1423, 1409, 1392, 1373, 1091, 981,952, 906, 860, 837 cm^−^^1^; Anal. Calcd. for C_23_H_15_Cl_2_N_5_O_4_S_4_: C 44.23%, H 2.42%, N 11.21%. Found: C 44.40%, H 2.28%, N 11.54%.

*N-(3-Chlorophenyl)-5-(4-chlorophenyl)-1,3,4-thiadiazole-2-sulfonamide* (**7i**). White powder, yield 37%, m.p. 258–260 °C; ^1^H-NMR (DMSO-*d*_6_): *δ* 11.75 (s, 1H, NH), 8.07 (d, 2H, ArH, *J* = 8.6 Hz), 7.66 (d, 2H, ArH, *J* = 8.6 Hz), 7.39 (t, 1H, ArH, *J* = 8.0 Hz), 7.31–7.30 (m, 1H, ArH), 7.26–7.21 (m, 2H, ArH); ^13^C-NMR (DMSO-*d*_6_): *δ* 172.0, 167.1, 137.9, 134.0, 131.7, 130.7, 130.2, 127.6, 125.8, 121.1, 120.0; IR: *ν* 3053 (NH), 3014 (CH-Ar), 2804, 1589, 1475, 1431, 1392, 1357, 1168, 1093, 985, 833 cm^−^^1^; Anal. Calcd. for C_14_H_9_Cl_2_N_3_O_2_S_2_: C 43.53%, H 2.35%, N 10.88%. Found: C 43.59%, H 2.25%, N 10.78%.

*Di-n-propyl(2-(4-chlorophenyl)-1,3,4-thiadiazole-5sulfonamido)-(phenyl)methylphosphonate* (**7j**). White solid, yield 34.1%, m.p. 203–205 °C; ^1^H-NMR (DMSO-*d*_6_) *δ*: 10.23 (s, 1H, NH), 7.92 (d, *J* = 8.6 Hz, 2H, ArH), 7.66 (d, *J* = 8.6 Hz, 2H, ArH), 7.34 (d, *J* = 7.4 Hz, 2H, ArH), 7.18 (t, 2H, ArH, *J* = 8.0 Hz), 7.13 (t, *J* = 7.4 Hz, 6.8 Hz, 1H, ArH), 4.97 (d, *J* = 29.1 Hz, 1H, CH), 3.88–3.98 (m, 2H, CH_2_), 3.75–3.81 (m, 1H, CH), 3.59–3.65 (m, 1H, CH), 1.53–1.60 (m, 2H, CH_2_), 1.36–1.43 (m, 2H, CH_2_), 0.84 (t, 3H, CH_3_, *J* = 7.4 Hz), 0.72 (t, 3H, CH_3_, *J* = 7.4 Hz); ^13^C-NMR (DMSO-*d*_6_): *δ* 171.3, 168.4, 137.6, 134.3, 130.3, 130.2, 128.9, 128.4, 128.3, 127.7, 68.8, 55.8, 54.6, 23.8, 23.6, 10.4.10.3; IR: *ν* 3064 (NH), 3015 (CH-Ar), 2966 (CH_3_), 2935, 2877, 1458, 1352, 1228, 1166, 1091, 1062, 1029, 1000, 923, 829, 694 cm^-1^; Anal. Calcd. for C_21_H_25_ClN_3_O_5_PS_2_: C 47.59%, H 4.75%, N 7.93%. Found: C 47.78%, H 4.30%, N 8.02%.

### 3.8. Antiviral biological assay

*Purification of TMV*. Using Gooding’s method [14], upper leaves of *Nicotiana tabacum L* inoculated with TMV were selected and ground in phosphate buffer, then filtered through a double layer pledget. The filtrate was centrifuged at 10,000 g, treated twice with PEG and centrifuged again. The whole experiment was carried out at 4 °C. Absorbance values were estimated at 260 nm using an ultraviolet spectrophotometer. 


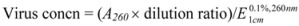


*Curative effect of compounds against TMV in vivo.* Growing leaves of *Nicotiana tabacum. L* of the same ages were selected. TMV (concentration of 6 × 10^−3 ^mg/mL) was dipped and inoculated on the whole leaves, then the leaves were washed with water and dried. The compound solution was smeared on the left side and the solvent was smeared on the right side for control. The local lesion numbers were then counted and recorded 3-4 d after inoculation [15]. For each compound, three repetitions were measured. The inhibition rate of the compound was then calculated according to the following formula (‘av’ means average):
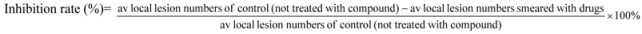


## 4. Conclusions

In the present study, we have described a mild, efficient and convenient method for the synthesis of 5-(4-chlorophenyl)-*N*-substituted-1,3,4-thiadiazole-2-sulfonamide derivatives from easily accessible *p*-chlorobenzoic acid. The incorporation of two different pharamacophores in a single structure led to the development of novel derivatives with moderate activity. Although, slightly less effective than the control agent ningnanmycin, compounds **7b** and **7i** showed promising anti-viral activity against TMV. Due to lack of structural diversity of the investigated compounds, a definite SAR could not be established and further research needs to be performed to identify lead structures with better activity.
